# A putative molecular network associated with colon cancer metastasis constructed from microarray data

**DOI:** 10.1186/s12957-017-1181-9

**Published:** 2017-06-19

**Authors:** Songtao Chu, Haipeng Wang, Miao Yu

**Affiliations:** 1 Department of Forensic Medicine of Basic Medical College, Beihua University, Jilin, 132013 Jilin Province China; 2 Gastrointestinal Colorectal and Anal Surgery, China-Japan Union Hospital of Jilin University, Changchun, 130000 Jilin Province China

**Keywords:** Colon cancer, Metastasis, Differentially expressed gene, Protein–protein interaction, Integrated network

## Abstract

**Background:**

This study aimed to identify the potential molecular network associated with colon cancer metastasis.

**Methods:**

A gene expression profile dataset (GSE40367) downloaded from Gene Expression Omnibus was used to identify and compare differentially expressed genes (DEGs) between primary colon adenocarcinoma tissues and matched tissue samples of liver metastases of colon adenocarcinoma. After the functional analysis of the DEGs, their protein–protein interactions (PPIs) were analyzed, and the transcription factors (TFs) and microRNAs (miRNAs) that regulated these DEGs were predicted. The data were used to construct an integrated network of DEGs, TFs, and miRNAs. Finally, the GSE68468 dataset was used to validate the DEGs associated with liver metastasis of colon adenocarcinoma identified in the GSE40367 dataset.

**Results:**

Compared with the primary colon adenocarcinoma sample, 262 DEGs were upregulated and 216 were downregulated in the liver metastasis sample. The DEGs were primarily involved in functions associated with cell junctions and cell adhesion. The DEGs included 17 genes encoding TFs, and 39 miRNAs that regulated DEGs were predicted. Further analysis of the DEGs led to the identification of 490 PPIs. The data were used to construct an integrated network consisting of DEGs, TFs, and miRNAs. DEGs with a high degree of connectivity in the network included *FGF2*, *ERBB4*, *PTPRC*, *CXCR4*, *CCL2*, and *CCL4*. The network also revealed that *FGF2* interacted with *ERBB4*, *PTPRC*, and *CXCR4* and that *PTPRC* interacted with *CXCR4*. Furthermore, *LCP2* and *APBB1IP* were predicted to target several other DEGs, including *PTPRC*, and miR-30a-3p and miR-30e-3p were predicted to regulate *ERBB4* and several other DEGs. Notably, *FGF2*, *ERBB4*, *PTPRC*, *LCP2*, *CCL2*, and *CCL4* were also identified as DEGs in the GSE68468 dataset.

**Conclusion:**

The DEGs, TFs, and miRNAs identified in this study might play key roles in colon cancer metastasis.

**Electronic supplementary material:**

The online version of this article (doi:10.1186/s12957-017-1181-9) contains supplementary material, which is available to authorized users.

## Background

Colon cancer is a potentially fatal disease that affects more than a quarter of a million people each year [1]. Despite recent advances in the diagnosis and treatment of colon cancer, there were still 95,270 estimated new cases of colon cancer and 49,190 estimated deaths associated with it in the USA in 2016 [2]. Tumor metastasis is the primary cause of disease recurrence and death in patients with colon cancer [3].

In recent years, remarkable advances have been made in the study of the molecular mechanisms underlying colon cancer metastasis. *CD44v6*, a gene required for the colon cancer cell migration and generation of metastatic colon tumors, has been identified as a functional biomarker and therapeutic target of colon cancer therapy, and low levels of *CD44v6* are associated with an increased probability of survival [3]. *CCAT2*, a novel long noncoding RNA transcript, is upregulated in microsatellite-stable colorectal cancer (CRC). *CCAT2* enhances tumor growth and metastasis via miR-17-5p, miR-20a, and MYC [4]. In addition, nuclear β-catenin is resistant to FOXO3a-mediated apoptosis and promotes colon cancer metastasis [5]. Galectin-3 mediates resistance to tumor necrosis factor-related apoptosis-inducing ligand (TRAIL) by inhibiting TRAIL binding to death receptors, thereby promoting the metastasis of colon adenocarcinoma cells [6]. MicroRNAs (miRNAs) have also been shown to be associated with colon cancer metastasis. For example, miR-200 has been shown to mediate epithelial-to-mesenchymal transition (EMT) and metastatic behavior in colon cancer [7, 8]. miR-192 is able to inhibit the metastasis of colon cancer to the liver by downregulating the expression of several target genes, including *Bcl-2*, *Zeb2*, and *VEGFA* [9]. However, despite these findings, the molecular mechanisms underlying the metastasis of colon cancer, especially in the context of colon adenocarcinoma, remain incompletely understood. In addition, approaches for analyzing the differences between primary tumor lesions and their matched distant metastases remain unclear.

The present study’s aim was to investigate the molecular mechanisms underlying colon cancer metastasis. For the same, we used publically available gene expression data from metastatic colon adenocarcinoma samples to identify and characterize genes that are differentially between primary colon adenocarcinoma tissues and matched liver metastasis tissues. The putative functions and protein–protein interactions (PPIs) associated with the differentially expressed genes (DEGs) were analyzed, and transcription factors (TFs) and miRNAs that regulated the DEGs were predicted. The goal of the study was to identify novel metastasis-related DEGs in colon adenocarcinoma and provide insights into the mechanisms underlying colon adenocarcinoma metastasis, which could potentially inform further studies.

## Methods

### Data source

Gene expression profile dataset GSE40367 [10] was extracted from the Gene Expression Omnibus (GEO) database (http://www.ncbi.nlm.nih.gov/geo/). The data were analyzed using Affymetrix Human Genome U133 Plus 2.0 Array (GPL570, Affymetrix, Santa Clara, CA, USA). The dataset included data from 61 primary and metastatic tumor specimens. Data from 14 colon adenocarcinoma samples, consisting of seven endothelial samples from primary colon adenocarcinoma tissues and seven tumor endothelial samples from matched liver metastases were extracted for further analysis.

Gene expression profile dataset GSE68468 was also downloaded from GEO and analyzed using Affymetrix Human Genome U133A Array (HG-U133A-GPL96). This dataset comprised data from several types of samples, including primary colon cancer, polyps, metastases, and matched normal mucosal samples. Data from 185 metastatic colon cancer tissues and 14 colon carcinoma liver metastases were used for data validation.

### Identification of DEGs

The Affymetrix CEL files were downloaded from GEO. The robust microarray analysis method [11] in the Affy package [12] was used for the initial processing of the data. This processing included background correction, quantile normalization, probe summarization, and translation of the probe ID to the gene symbol. Empirical Bayes statistics in LIMMA (Linear Models for Microarray Data, http://www.bioconductor.org/packages/release/bioc/html/limma.html) package in R (Version 3.0.0) provided by bioconductor software (http://bioconductor.org/help/search/index.html?q=R+software+/) [13] was utilized to calculate the significance (*P* value) of the differences in expression of the DEGs between the primary tumor tissues and the matched metastatic tissues. A *P* value of <0.05 was selected as the cutoff criterion for defining DEGs.

### Functional enrichment analysis

The online tool called Database for Annotation, Visualization, and Integrated Discovery, (DAVID; version 6.7; http://david.abcc.ncifcrf.gov/) [14] was used to conduct the Gene Ontology (GO) functional enrichment analysis for cellular components (CC), biological processes (BPs), molecular functions (MFs), and the KEGG (Kyoto Encyclopedia of Genes and Genomes) pathway analysis. A P value of <0.01(calculated using Fisher’s exact test) and gene count >2 were set as the cutoff criteria for the GO functional enrichment analysis. A *P* value of <0.05 and gene count of >2 were chosen as the cutoff criteria for the KEGG pathway enrichment analysis.

### PPI network construction

PPIs among the DEGs were identified using the STRING (Search Tool for the Retrieval of Interacting Genes; http://string-db.org/) database, which integrates a large number of known and predicted protein interactions [15]. PPIs with a combined score of >0.4 were used to construct the PPI network, and the network was visualized using Cytoscape (http://cytoscape.org/) [16]. Network modules were extracted from the original PPI network based on MCODE analysis [17]. The default parameters (K-Core, 2; degree cutoff, 2; max. depth, 100; node score cutoff, 0.2) were used as the cutoff criteria for the identification of network modules. The GO functional and KEGG pathway enrichment analyses of genes in the modules were conducted using the threshold values described above.

### Prediction of DEG regulators

TFs that regulate the DEGs identified were predicted using integrated TF platform (ITFP), which contains a large number of mammalian TFs and their targets [18].

miRNAs that regulate the DEGs identified were analyzed using WEB-based gene set analysis toolkit (Webgestalt; http://www.webgestalt.org) [19]. The threshold number of genes targeted by a regulatory miRNA was defined as ≥4. The threshold *P* value (calculated by Fisher’s exact test) was set at <0.01.

### Construction of an integrated network

The integrated network consisting of PPIs, TF-DEG pairs, and miRNA-DEG pairs was visualized using Cytoscape. The connectivity degree of the nodes in the network was calculated according to the scale-free property of the network.

### Data validation

Dataset GSE68468 from the GEO database was used to validate the DEGs identified in GSE40367. The DEGs were screened using the same method and threshold values. DEGs common to both datasets were determined using a Venn diagram.

## Results

### Identification and characterization of DEGs

**Fig. 1:**
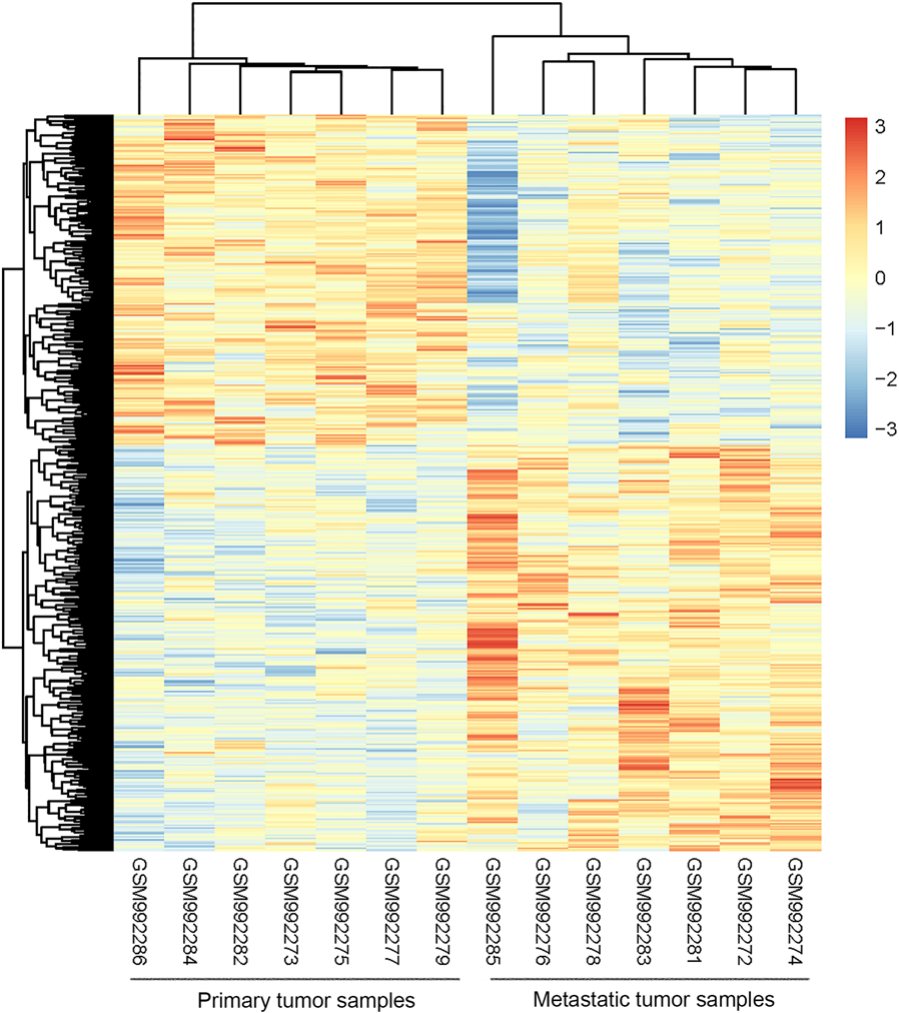
A heatmap of DEGs between primary colon adenocarcinoma tissues and liver metastases. Each *row* represents a single gene, and each *column* represents a sample. The *labels* below represent the sample number in the dataset. *Red* represents upregulation, and *blue* represents downregulation

Compared with the primary samples, 262 genes were upregulated and 216 genes were downregulated in the metastasis samples. The hierarchy cluster analysis of the DEGs was able to distinguish the two groups of samples (Fig. 1), confirming the reliability of the results for subsequent analysis.

**Fig. 2:**
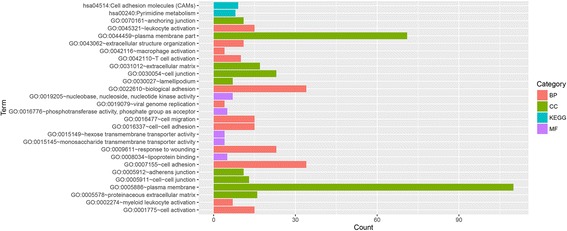
The results of the GO and pathway enrichment analyses of the DEGs. *BP* biological process, *CC* cellular component, *MF* molecular function, *KEGG* Kyoto Encyclopedia of Genes and Genomes, *GO* Gene Ontology

The biological functions of the DEGs were analyzed using GO and KEGG pathway functional enrichment analyses. The DEGs were predicted to be significantly associated with pathways related to cell adhesion molecules (*P* = 0.03) and pyrimidine metabolism (*P* = 0.03), as well as several GO functions, including leukocyte activation (*P* = 0.002), extracellular structure organization (*P* = 0.005), cell junctions (*P* = 0.001), and cell adhesion (*P* = 0.00015; Fig. 2) (Additional file 1).

### Analysis of the PPI network and modules

**Fig. 3:**
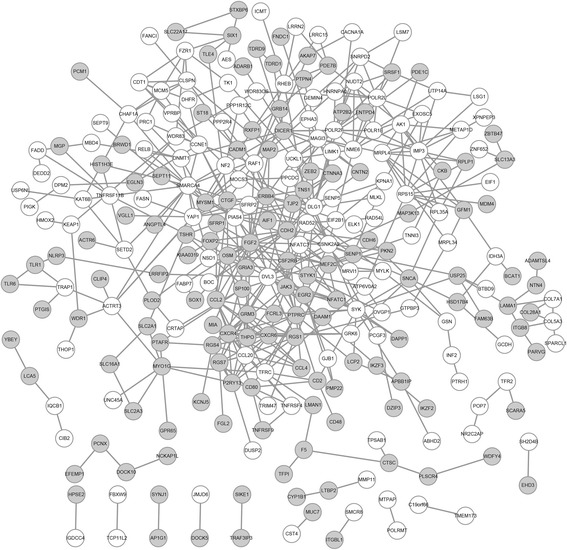
The PPI network of DEGs. *Gray nodes* represent upregulated genes, and *white nodes* represent down regulated genes. *Nodes* represent proteins, and *lines* represent PPI pairs. The degree of connectivity of each node reflects the number of nodes that interact with it

Putative PPIs associated with the DEGs were investigated PPIs using STRING. A total of 490 PPIs involving 70 DEGs were identified (Fig. 3). According to the PPI network, *FGF2* interacted with *ERBB4*, *PTPRC*, and *CXCR4* and *PTPRC* interacted with *CXCR4*.

**Fig. 4:**
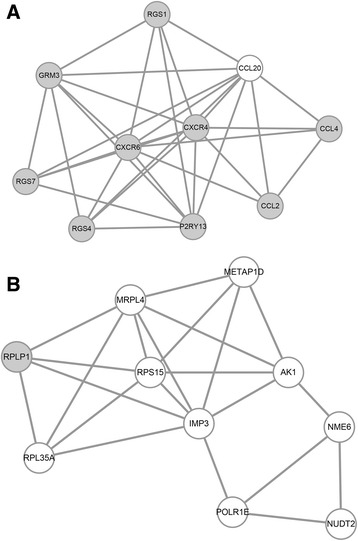
Modules identified in the PPI network of DEGs. **a** Module 1 and **b** module 2. *Gray nodes* represent upregulated genes, and *white nodes* represent downregulated genes

A total of 10 modules in the PPI network met the cutoff criteria. The two modules (modules 1 and 2) with the highest scores and several additional nodes were selected for further analysis. Module 1 included 10 DEGs (nine upregulated genes and one downregulated gene), including *CXCR4*, *CXCR6*, *C-C Motif Chemokine Ligand 2* (*CCL2)*, *CCL4*, and *CCL20* (Fig. 4a). Module 2 also comprised 10 DEGs (one upregulated gene and nine downregulated genes), including *RPLP1*, *RPL35A*, *RPS15*, and *MRPL4* (Fig. 4b).

**Fig. 5:**
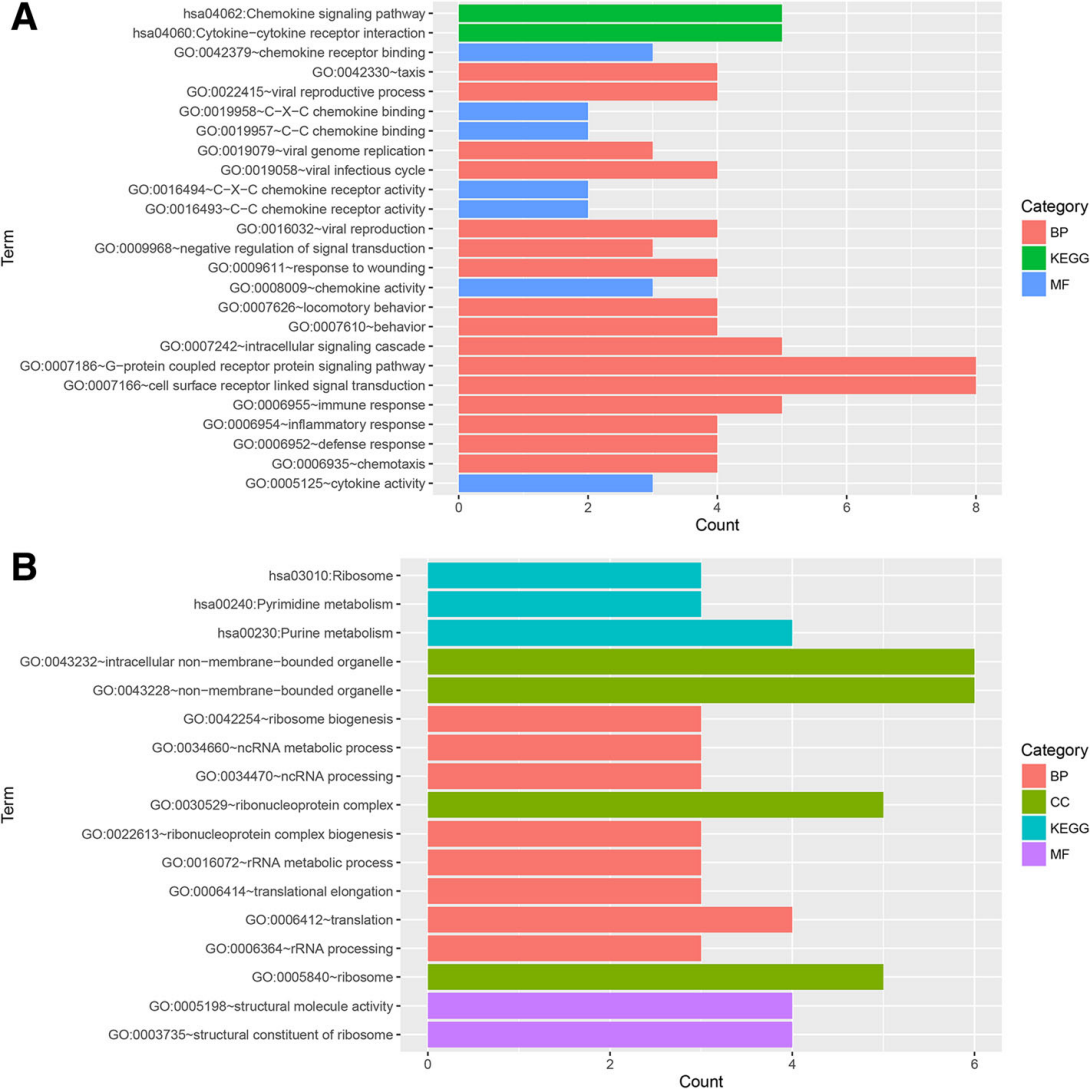
The results of the GO and pathway enrichment analyses of DEGs in module 1 (**a**) and module 2 (**b**). *BP* biological process, *CC* cellular component, *MF* molecular function, *KEGG* Kyoto Encyclopedia of Genes and Genomes, *GO* Gene Ontology

The functional enrichment analysis revealed that DEGs in module 1 were significantly enriched for factors associated with chemokine signaling (*P* = 0.000025) and cytokine–cytokine receptor interactions (*P* = 0.000095), as well as several GO functions, including cell surface receptor linked signal transduction (*P* = 0.00015), G-protein coupled receptor protein signaling (*P* = 0.03), and immune responses (*P* = 0.00068; Fig. 5a). DEGs in module 2 were markedly enriched for pathways associated with ribosomes (*P* = 0.00011) and pyrimidine and purine metabolism (*P* = 0.0049), as well as several GO functions, including ncRNA metabolism (*P* = 0.005) and ribonucleoprotein complex biogenesis and translation (*P* = 0.004; Fig. 5b) (Additional file 1).

### Prediction of regulatory TFs and miRNAs

We also investigated TFs and miRNAs that potentially regulate the identified DEGs. Seventeen DEGs encoded TFs that targeted other DEGs. Five DEGs encoding TFs (*ADARB1*, *APBB1IP*, *ARHGAP25*, *LCP2*, and *MCM5*) that targeted more than five genes (Table 1) were selected for further analysis. A total of 39 miRNAs predicted to regulate the DEGs met the cutoff criteria. The 17 miRNAs associated with the 10 lowest *P* values (e.g., miR-506, miR-330, miR-17-5P, and miR-124A) targeted a total of 88 DEGs (Table 2).

**Table 1:** The transcription factors targeting more than five differentially expressed genes

TF	Target	Score	TF	Target	Score
*ADARB1*	*ATP2B2*	0.0431271	*LCP2*	*AIF1*	0.0485591
*ADARB1*	*C17orf51*	0.0453939	*LCP2*	*APBB1IP*	0.0500103
*ADARB1*	*ICMT*	0.0576544	*LCP2*	*CCL4*	0.0490895
*ADARB1*	*LMAN1*	0.0474452	*LCP2*	*CSF2RB*	0.0501211
*ADARB1*	*RPLP1*	0.0452993	*LCP2*	*EVI2B*	0.0544675
*ADARB1*	*SULT1C2*	0.0493526	*LCP2*	*MNDA*	0.0509129
*APBB1IP*	*AIF1*	0.0558902	*LCP2*	*MS4A6A*	0.0476156
*APBB1IP*	*CD48*	0.0580736	*LCP2*	*PARVG*	0.0486343
*APBB1IP*	*LCP2*	0.0500103	*LCP2*	*PTPRC*	0.0510363
*APBB1IP*	*MYO1G*	0.0496366	*MCM5*	*CDT1*	0.0528149
*APBB1IP*	*NCKAP1L*	0.0529768	*MCM5*	*CHAF1A*	0.0556453
*APBB1IP*	*PTPRC*	0.0576564	*MCM5*	*DNMT1*	0.0530079
*ARHGAP25*	*OR5L2*	0.0474039	*MCM5*	*FANCI*	0.0524277
*ARHGAP25*	*PARVG*	0.0495445	*MCM5*	*LRRFIP2*	0.0442217
*ARHGAP25*	*PTAFR*	0.0442268	*MCM5*	*PRC1*	0.0563632
*ARHGAP25*	*PTPRC*	0.0448310	*MCM5*	*RAD54L*	0.0507543
*ARHGAP25*	*SYK*	0.0486975	*MCM5*	*SPARCL1*	0.0495283
*ARHGAP25*	*WDFY4*	0.0452244	*MCM5*	*TK1*	0.0516265

*TF* transcription factor

**Table 2:** The top 10 results of predicted microRNAs with a lower *P* value

MicroRNA	Target gene count	Adjusted *P* value
hsa_GTGCCTT, miR-506	22	0.0004
hsa_TGCTTTG, miR-330	14	0.0004
hsa_GCACTTT, miR-17-5P, miR-20A, miR-106A, miR-106B, miR-20B, miR-519D	20	0.0004
hsa_TGCCTTA, miR-124A	18	0.0005
hsa_ACTGAAA, miR-30A-3P, miR-30E-3P	10	0.0006
hsa_ATACTGT, miR-144	10	0.0006
hsa_AATGTGA, miR-23A, miR-23B	15	0.0006
hsa_ACTGTAG, miR-139	8	0.0006
hsa_AGCATTA, miR-155	8	0.0008
hsa_GCATTTG, miR-105	9	0.0008

### Analysis of the integrated network

**Fig. 6:**
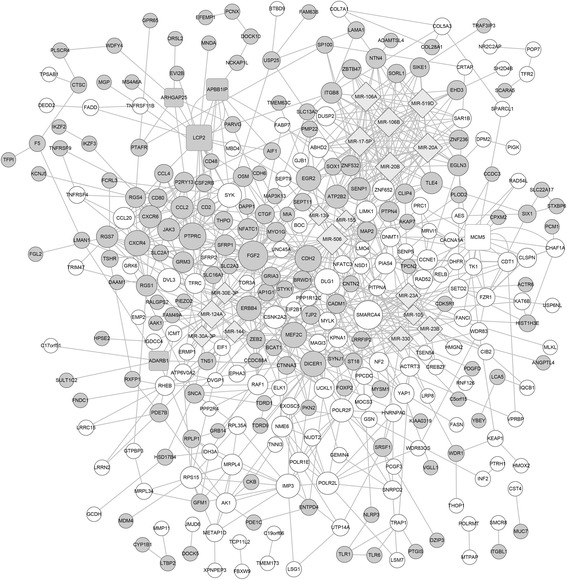
The integrated network consisting of DEGs, TFs, and miRNAs. *Round* and *rectangular gray nodes* represent upregulated genes, and *round* and *rectangular white nodes* represent downregulated genes. The *rectangular nodes* represent TFs, and *diamonds* represent miRNAs

The PPIs, TF-DEG pairs, and miRNA-DEG pairs were used to construct an integrated network comprising 323 nodes and 785 relation pairs (Fig. 6). miRNAs and DEGs with the highest degree of connectivity included miR-506 (degree = 22), *FGF2* (degree = 20), miR-106A (degree = 20), and *SMARCA4* (degree = 19). The degree of the TF-encoding genes *LCP2* and *MCM5* was 15 (Table 3). In addition, the network revealed that *LCP2* and *APBB1IP* targeted several DEGs, including *PTPRC*, and that miR-30a-3p and miR-30e-3p regulated several DEGs, including *ERBB4*.

**Table 3:** The nodes with a degree at least 10 in the integrated network

Node	Degree	Node	Degree	Node	Degree	Node	Degree
miR-506	22	*CDH2*	16	miR-330	14	*SENP1*	11
*FGF2*	20	*CXCR4*	15	*IMP3*	13	*APBB1IP*	10
miR-106A	20	*DICER1*	15	*SYK*	13	*CCL20*	10
miR-106B	20	*DLG1*	15	*ATP2B2*	12	miR-144	10
miR-17-5P	20	*LCP2*	15	*DVL3*	12	miR-30A-3P	10
miR-20A	20	*MCM5*	15	*POLR2F*	12	miR-30E-3P	10
miR-20B	20	*MEF2C*	15	*CCL2*	11	*RGS7*	10
miR-519D	20	miR-23A	15	*CSNK2A2*	11	*RPS15*	10
*SMARCA4*	19	miR-23B	15	*CXCR6*	11	*TLE4*	10
miR-124A	18	*EGR2*	14	*JAK3*	11	*ZNF652*	10
*PTPRC*	17	*ERBB4*	14	*POLR2L*	11		

### Data validation of the DEGs

**Fig. 7:**
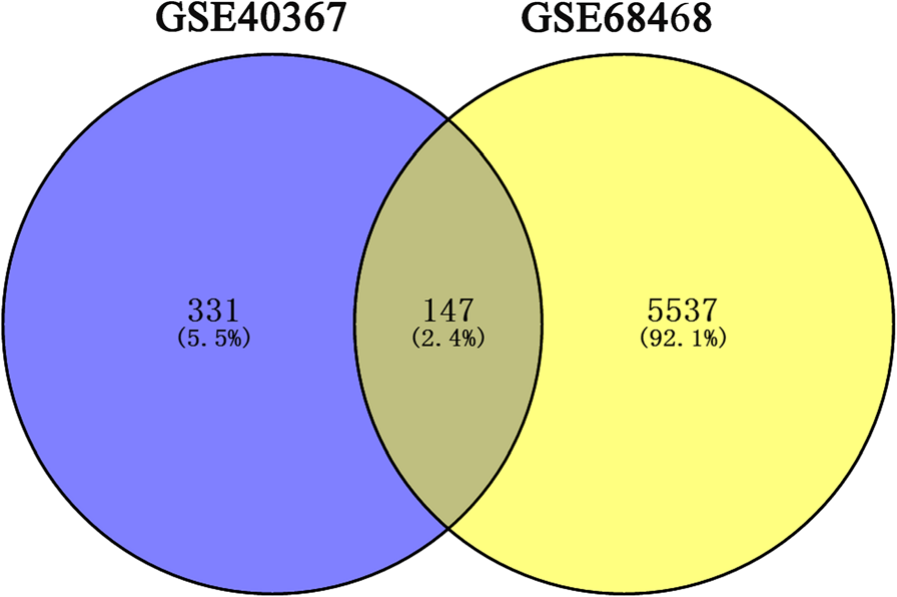
The Venn diagram showing the overlapped genes in both GSE40367 dataset and GSE64486 dataset

A total of 5537 DEGs were identified in the comparison of non-metastatic colon carcinoma samples and metastasis samples from the GSE68468 dataset. Among them, 147 genes (e.g., *FGF2*, *ERBB4*, *PTPRC*, *LCP2*, *MCM5*, *CCL2*, *CCL4*, *RPL35A*, and *MRPL4*) were identified in both GSE40367 and GSE68468 (Fig. 7).

## Discussion

The present study identified 262 genes that were upregulated, and 216 genes were downregulated in liver metastasis samples compared with matched primary colon adenocarcinoma samples. In the integrated network constructed from these data, DEGs with the highest degree of connectivity included *FGF2*, *ERBB4*, *PTPRC*, and *CXCR4. FGF2* was predicted to interact with *ERBB4*, *PTPRC*, *CXCR4*, *CCL2*, and *CCL4*, whereas *PTPRC* was predicted to interact with *CXCR4*.

*FGF2* encodes fibroblast growth factor 2, a protein with mitogenic and angiogenic activities, which contributes to tumor growth [20]. *FGF2* is highly expressed in metastatic CRC [21], consistent with the results of the present study. *FGF2* promotes CRC cell migration and invasion via integrin αvβ5-mediated adhesion and FGF receptor-SRC signaling [22]. The IC50 of 5-fluorouracil (5-FU) decreases in cells deficient for *FGF2* compared with control CRC cells in vitro [23]. Moreover, high *FGF2* expression levels are correlated with a lower response rate to 5-FU and overall survival in CRC patients [23]. These results indicate that *FGF2* plays an important role in colon cancer metastasis. In this study, *FGF2* was predicted to interact with *ERBB4*, *PTPRC*, *CXCR4*, *CCL2*, and *CCL4. ERBB4* encodes Erb-B2 receptor tyrosine kinase 4, a member of the epidermal growth factor receptor subfamily. *ERBB4* is overexpressed in human colon cancer and promotes cellular transformation [24]. A previous study found that *ErbB4* and the metastasis-enhancing gene *KAI1 C-terminal interacting tetraspanin* (*KITENIN*) can upregulate c-Jun and promote CRC cell invasion [25]. *ERBB4* was predicted to be regulated by miR-30a-3p and miR-30e-3p. A previous study reported that miR-30A inhibits EMT in lung cancer [26], suggesting that low levels of miR-30A might enhance EMT in cancer. *PTPRC* encodes a member of the protein tyrosine phosphatase (PTP) family, which comprises proteins commonly activated in tumors [27]. Consistent with the results of the present study, *CXCR4* (*C-X-C motif chemokine receptor 4*) is highly expressed in metastatic colon cancer in the liver compared with primary colon cancer tissue, and elevated *CXCR4* expression levels contribute to poor survival [28–30]. *CCL2* is upregulated in metastatic CRC and functions as a prognostic marker of liver metastasis due its role in recruiting myeloid cells [31]. *CCL4* has also been shown to play a crucial role in metastatic CRC via interactions with the receptor CCR5 [32, 33]. No other studies have reported an association between *PTPRC* and colon cancer metastasis. However, the PTP family member *PRL-3* is associated with CRC metastasis to the liver and CRC prognosis [34, 35]. As *PTPRC* was predicted to interact with *CXCR4*, we speculated that *PTPRC* may also be involved in the metastasis of colon cancer to the liver. In this study, *PTPRC* was predicted to be targeted by TFs encoded by the upregulated genes *APBB1IP* and *LCP2. APBB1IP* encodes a Rap1-GTP-interacting adaptor molecule. Rap1-GTPase activation mediates breast cancer cell migration [36], and activated *Rap1* can promote prostate cancer metastasis [37]. *LCP2*, also referred to as *SLP-76*, promotes T-cell development and activation [38]. To date, no other studies have reported an association between *APBB1IP* and *LCP2.* However, both genes targeted *PTPRC*, and *PTPRC* interacted with *FGF2.* The aforementioned studies indicate that *FGF2* plays an important role in colon cancer metastasis. Therefore, *APBB1IP*, *LCP2*, and *PTPRC* might also play a role in colon cancer metastasis.

Despite the significance of our findings, several limitations to this study are worth noting. The results are solely predictions; therefore, they should be confirmed by laboratory data. Furthermore, our findings should be confirmed in a larger sample size. The expression patterns of the genes identified in the present study should be validated by large-scale studies in the future. Furthermore, the interactions among the DEGs identified and their relationship with the predicted regulatory TFs and miRNAs should be confirmed.

## Conclusions

In conclusion, we identified 262 upregulated and 216 downregulated DEGs in liver metastases originating from colon adenocarcinoma and used these data to construct a network of DEGs, regulatory TFs, and miRNAs. Genes that played a prominent role in this network included *FGF2*, *ERBB4*, *PTPRC*, *CXCR4*, *CCL2*, and *CCL4.* The set of DEGs also comprised genes encoding TFs, including *APBB1IP* and *LCP2*, and miRNAs, including miR-30a-3p and miR-30e-3p. This is the first evidence supporting a role for *PTPRC*, *APBB1IP*, *LCP2*, miR-30a-3p, and miR-30e-3p in colon cancer metastasis. These findings might provide new information that can serve as the basis for future experimental studies.

## Additional file

Additional file 1: Result of functional enrichment analysis. (XLSX 24 kb)

## Abbreviations

BP: Biological process
CC: Cellular component
CRC: Colorectal cancer
DEGs: Differentially expressed genes
EMT: Epithelial-to-mesenchymal transition
GEO: Gene Expression Omnibus
GO: Gene Ontology
ITFP: Integrated TF platform
KEGG: Kyoto Encyclopedia of Genes and Genomes
MF: Molecular function
miRNAs: MicroRNAs
PPIs: Protein–protein interactions
PTP: Protein tyrosine phosphatase
TFs: Transcription factors
